# Supplementary material for: A fault-intrusion-tolerant system and deadline-aware algorithm for scheduling scientific workflow in the cloud
Source: PeerJ Comput Sci. 2021 Nov 2;7:e747. doi: 10.7717/peerj-cs.747 (PMC8576554; doi:10.7717/peerj-cs.747)
Supplement: Supplemental Information 6 [file peerj-cs-07-747-s006.zip › Myproject1.1/docs/org/cloudbus/cloudsim/class-use/Cloudlet.html]

Uses of Class org.cloudbus.cloudsim.Cloudlet


JavaScript is disabled on your browser.


- Overview
- Package
- Class
- Use
- Tree
- Deprecated
- Index
- Help

- Prev
- Next

- Frames
- No Frames

- All Classes

## Uses of Class org.cloudbus.cloudsim.Cloudlet

- Packages that use Cloudlet

  | Package | Description |
  | --- | --- |
  |  |  |
  | --- | --- |
  | org.cloudbus.cloudsim |  |
  | org.cloudbus.cloudsim.examples |  |
  | org.cloudbus.cloudsim.examples.power |  |
  | org.cloudbus.cloudsim.examples.power.planetlab |  |
  | org.cloudbus.cloudsim.examples.power.random |  |
  | org.cloudbus.cloudsim.lists |  |
  | org.cloudbus.cloudsim.network.datacenter |  |
  | org.cloudbus.cloudsim.util |  |
  | org.workflowsim |  |
  | org.workflowsim.scheduler |  |
  | org.workflowsim.utils |  |
- - ### Uses of Cloudlet in org.cloudbus.cloudsim

    Fields in org.cloudbus.cloudsim with type parameters of type Cloudlet

    | Modifier and Type | Field and Description |
    | --- | --- |
    |  |  |
    | --- | --- |
    | `protected java.util.List<? extends Cloudlet>` | DatacenterBroker.`cloudletList` The cloudlet list. |
    | `protected java.util.List<? extends Cloudlet>` | DatacenterBroker.`cloudletReceivedList` The cloudlet received list. |
    | `protected java.util.List<? extends Cloudlet>` | DatacenterBroker.`cloudletSubmittedList` The cloudlet submitted list. |

    Methods in org.cloudbus.cloudsim with type parameters of type Cloudlet

    | Modifier and Type | Method and Description |
    | --- | --- |
    |  |  |
    | --- | --- |
    | `<T extends Cloudlet>  java.util.List<T>` | DatacenterBroker.`getCloudletList()` Gets the cloudlet list. |
    | `<T extends Cloudlet>  java.util.List<T>` | DatacenterBroker.`getCloudletReceivedList()` Gets the cloudlet received list. |
    | `<T extends Cloudlet>  java.util.List<T>` | DatacenterBroker.`getCloudletSubmittedList()` Gets the cloudlet submitted list. |
    | `protected <T extends Cloudlet>  void` | DatacenterBroker.`setCloudletList(java.util.List<T> cloudletList)` Sets the cloudlet list. |
    | `protected <T extends Cloudlet>  void` | DatacenterBroker.`setCloudletReceivedList(java.util.List<T> cloudletReceivedList)` Sets the cloudlet received list. |
    | `protected <T extends Cloudlet>  void` | DatacenterBroker.`setCloudletSubmittedList(java.util.List<T> cloudletSubmittedList)` Sets the cloudlet submitted list. |

    Methods in org.cloudbus.cloudsim that return Cloudlet

    | Modifier and Type | Method and Description |
    | --- | --- |
    |  |  |
    | --- | --- |
    | `abstract Cloudlet` | CloudletScheduler.`cloudletCancel(int clId)` Cancels execution of a cloudlet. |
    | `Cloudlet` | CloudletSchedulerSpaceShared.`cloudletCancel(int cloudletId)` Cancels execution of a cloudlet. |
    | `Cloudlet` | CloudletSchedulerTimeShared.`cloudletCancel(int cloudletId)` Cancels execution of a cloudlet. |
    | `Cloudlet` | ResCloudlet.`getCloudlet()` Gets this Cloudlet object. |
    | `abstract Cloudlet` | CloudletScheduler.`getNextFinishedCloudlet()` Returns the next cloudlet in the finished list, $null if this list is empty. |
    | `Cloudlet` | CloudletSchedulerSpaceShared.`getNextFinishedCloudlet()` Returns the next cloudlet in the finished list, $null if this list is empty. |
    | `Cloudlet` | CloudletSchedulerTimeShared.`getNextFinishedCloudlet()` Returns the next cloudlet in the finished list, $null if this list is empty. |
    | `abstract Cloudlet` | CloudletScheduler.`migrateCloudlet()` Returns one cloudlet to migrate to another vm. |
    | `Cloudlet` | CloudletSchedulerSpaceShared.`migrateCloudlet()` Returns one cloudlet to migrate to another vm. |
    | `Cloudlet` | CloudletSchedulerTimeShared.`migrateCloudlet()` Returns one cloudlet to migrate to another vm. |

    Methods in org.cloudbus.cloudsim with parameters of type Cloudlet

    | Modifier and Type | Method and Description |
    | --- | --- |
    |  |  |
    | --- | --- |
    | `abstract double` | CloudletScheduler.`cloudletSubmit(Cloudlet gl)` Receives an cloudlet to be executed in the VM managed by this scheduler. |
    | `double` | CloudletSchedulerDynamicWorkload.`cloudletSubmit(Cloudlet cl)` Receives an cloudlet to be executed in the VM managed by this scheduler. |
    | `double` | CloudletSchedulerSpaceShared.`cloudletSubmit(Cloudlet cloudlet)` |
    | `double` | CloudletSchedulerTimeShared.`cloudletSubmit(Cloudlet cloudlet)` |
    | `abstract double` | CloudletScheduler.`cloudletSubmit(Cloudlet gl, double fileTransferTime)` Receives an cloudlet to be executed in the VM managed by this scheduler. |
    | `double` | CloudletSchedulerDynamicWorkload.`cloudletSubmit(Cloudlet cl, double fileTransferTime)` Receives an cloudlet to be executed in the VM managed by this scheduler. |
    | `double` | CloudletSchedulerSpaceShared.`cloudletSubmit(Cloudlet cloudlet, double fileTransferTime)` Receives an cloudlet to be executed in the VM managed by this scheduler. |
    | `double` | CloudletSchedulerTimeShared.`cloudletSubmit(Cloudlet cloudlet, double fileTransferTime)` Receives an cloudlet to be executed in the VM managed by this scheduler. |

    Method parameters in org.cloudbus.cloudsim with type arguments of type Cloudlet

    | Modifier and Type | Method and Description |
    | --- | --- |
    |  |  |
    | --- | --- |
    | `void` | DatacenterBroker.`submitCloudletList(java.util.List<? extends Cloudlet> list)` This method is used to send to the broker the list of cloudlets. |

    Constructors in org.cloudbus.cloudsim with parameters of type Cloudlet

    Constructor and Description

    `ResCloudlet(Cloudlet cloudlet)`

    Allocates a new ResCloudlet object upon the arrival of a Cloudlet object.

    `ResCloudlet(Cloudlet cloudlet,
    long startTime,
    int duration,
    int reservID)`

    Allocates a new ResCloudlet object upon the arrival of a Cloudlet object.
  - ### Uses of Cloudlet in org.cloudbus.cloudsim.examples

    Methods in org.cloudbus.cloudsim.examples that return types with arguments of type Cloudlet

    | Modifier and Type | Method and Description |
    | --- | --- |
    |  |  |
    | --- | --- |
    | `java.util.List<Cloudlet>` | CloudSimExample8.GlobalBroker.`getCloudletList()` |

    Method parameters in org.cloudbus.cloudsim.examples with type arguments of type Cloudlet

    | Modifier and Type | Method and Description |
    | --- | --- |
    |  |  |
    | --- | --- |
    | `protected void` | CloudSimExample8.GlobalBroker.`setCloudletList(java.util.List<Cloudlet> cloudletList)` |
  - ### Uses of Cloudlet in org.cloudbus.cloudsim.examples.power

    Fields in org.cloudbus.cloudsim.examples.power with type parameters of type Cloudlet

    | Modifier and Type | Field and Description |
    | --- | --- |
    |  |  |
    | --- | --- |
    | `protected static java.util.List<Cloudlet>` | RunnerAbstract.`cloudletList` The cloudlet list. |

    Method parameters in org.cloudbus.cloudsim.examples.power with type arguments of type Cloudlet

    | Modifier and Type | Method and Description |
    | --- | --- |
    |  |  |
    | --- | --- |
    | `static void` | Helper.`printCloudletList(java.util.List<Cloudlet> list)` Prints the Cloudlet objects. |
  - ### Uses of Cloudlet in org.cloudbus.cloudsim.examples.power.planetlab

    Methods in org.cloudbus.cloudsim.examples.power.planetlab that return types with arguments of type Cloudlet

    | Modifier and Type | Method and Description |
    | --- | --- |
    |  |  |
    | --- | --- |
    | `static java.util.List<Cloudlet>` | PlanetLabHelper.`createCloudletListPlanetLab(int brokerId, java.lang.String inputFolderName)` Creates the cloudlet list planet lab. |
  - ### Uses of Cloudlet in org.cloudbus.cloudsim.examples.power.random

    Methods in org.cloudbus.cloudsim.examples.power.random that return types with arguments of type Cloudlet

    | Modifier and Type | Method and Description |
    | --- | --- |
    |  |  |
    | --- | --- |
    | `static java.util.List<Cloudlet>` | RandomHelper.`createCloudletList(int brokerId, int cloudletsNumber)` Creates the cloudlet list. |
  - ### Uses of Cloudlet in org.cloudbus.cloudsim.lists

    Methods in org.cloudbus.cloudsim.lists with type parameters of type Cloudlet

    | Modifier and Type | Method and Description |
    | --- | --- |
    |  |  |
    | --- | --- |
    | `static <T extends Cloudlet>  T` | CloudletList.`getById(java.util.List<T> cloudletList, int id)` Gets the by id. |
    | `static <T extends Cloudlet>  int` | CloudletList.`getPositionById(java.util.List<T> cloudletList, int id)` Returns the position of the cloudlet with that id, if it exists. |
    | `static <T extends Cloudlet>  void` | CloudletList.`sort(java.util.List<T> cloudletList)` Sorts the Cloudlets in a list based on their lengths. |
  - ### Uses of Cloudlet in org.cloudbus.cloudsim.network.datacenter

    Subclasses of Cloudlet in org.cloudbus.cloudsim.network.datacenter

    | Modifier and Type | Class and Description |
    | --- | --- |
    |  |  |
    | --- | --- |
    | `class` | `NetworkCloudlet` NetworkCloudlet class extends Cloudlet to support simulation of complex applications. |

    Methods in org.cloudbus.cloudsim.network.datacenter with type parameters of type Cloudlet

    | Modifier and Type | Method and Description |
    | --- | --- |
    |  |  |
    | --- | --- |
    | `<T extends Cloudlet>  java.util.List<T>` | NetDatacenterBroker.`getCloudletReceivedList()` Gets the cloudlet received list. |
    | `<T extends Cloudlet>  java.util.List<T>` | NetDatacenterBroker.`getCloudletSubmittedList()` Gets the cloudlet submitted list. |
    | `protected <T extends Cloudlet>  void` | NetDatacenterBroker.`setCloudletReceivedList(java.util.List<T> cloudletReceivedList)` Sets the cloudlet received list. |
    | `protected <T extends Cloudlet>  void` | NetDatacenterBroker.`setCloudletSubmittedList(java.util.List<T> cloudletSubmittedList)` Sets the cloudlet submitted list. |

    Methods in org.cloudbus.cloudsim.network.datacenter that return Cloudlet

    | Modifier and Type | Method and Description |
    | --- | --- |
    |  |  |
    | --- | --- |
    | `Cloudlet` | NetworkCloudletSpaceSharedScheduler.`cloudletCancel(int cloudletId)` Cancels execution of a cloudlet. |
    | `Cloudlet` | NetworkCloudletSpaceSharedScheduler.`getNextFinishedCloudlet()` Returns the next cloudlet in the finished list, $null if this list is empty. |
    | `Cloudlet` | NetworkCloudletSpaceSharedScheduler.`migrateCloudlet()` Returns one cloudlet to migrate to another vm. |

    Methods in org.cloudbus.cloudsim.network.datacenter with parameters of type Cloudlet

    | Modifier and Type | Method and Description |
    | --- | --- |
    |  |  |
    | --- | --- |
    | `double` | NetworkCloudletSpaceSharedScheduler.`cloudletSubmit(Cloudlet cloudlet)` |
    | `double` | NetworkCloudletSpaceSharedScheduler.`cloudletSubmit(Cloudlet cloudlet, double fileTransferTime)` Receives an cloudlet to be executed in the VM managed by this scheduler. |
  - ### Uses of Cloudlet in org.cloudbus.cloudsim.util

    Methods in org.cloudbus.cloudsim.util that return types with arguments of type Cloudlet

    | Modifier and Type | Method and Description |
    | --- | --- |
    |  |  |
    | --- | --- |
    | `java.util.ArrayList<Cloudlet>` | WorkloadFileReader.`generateWorkload()` Reads job information from a given file. |
    | `java.util.List<Cloudlet>` | WorkloadModel.`generateWorkload()` Returns a list with the jobs generated by the workload. |
  - ### Uses of Cloudlet in org.workflowsim

    Subclasses of Cloudlet in org.workflowsim

    | Modifier and Type | Class and Description |
    | --- | --- |
    |  |  |
    | --- | --- |
    | `class` | `Job` Job is an extention to Task. |
    | `class` | `Task` Task is an extention to Cloudlet in CloudSim. |

    Fields in org.workflowsim with type parameters of type Cloudlet

    | Modifier and Type | Field and Description |
    | --- | --- |
    |  |  |
    | --- | --- |
    | `protected java.util.List<? extends Cloudlet>` | WorkflowEngine.`jobsList` The job list. |
    | `protected java.util.List<? extends Cloudlet>` | WorkflowEngine.`jobsReceivedList` The job received list. |
    | `protected java.util.List<? extends Cloudlet>` | WorkflowEngine.`jobsSubmittedList` The job submitted list. |

    Methods in org.workflowsim with type parameters of type Cloudlet

    | Modifier and Type | Method and Description |
    | --- | --- |
    |  |  |
    | --- | --- |
    | `<T extends Cloudlet>  java.util.List<T>` | WorkflowEngine.`getJobsList()` Gets the job list. |
    | `<T extends Cloudlet>  java.util.List<T>` | WorkflowEngine.`getJobsReceivedList()` Gets the job received list. |
    | `<T extends Cloudlet>  java.util.List<T>` | WorkflowEngine.`getJobsSubmittedList()` Gets the job submitted list. |

    Methods in org.workflowsim with parameters of type Cloudlet

    | Modifier and Type | Method and Description |
    | --- | --- |
    |  |  |
    | --- | --- |
    | `protected double` | DatacenterExtended.`processDataStageIn(java.util.List<File> requiredFiles, Cloudlet cl)` |

    Method parameters in org.workflowsim with type arguments of type Cloudlet

    | Modifier and Type | Method and Description |
    | --- | --- |
    |  |  |
    | --- | --- |
    | `void` | WorkflowEngine.`submitCloudletList(java.util.List<? extends Cloudlet> list)` This method is used to send to the broker the list of cloudlets. |
  - ### Uses of Cloudlet in org.workflowsim.scheduler

    Methods in org.workflowsim.scheduler with parameters of type Cloudlet

    | Modifier and Type | Method and Description |
    | --- | --- |
    |  |  |
    | --- | --- |
    | `protected double` | DataAwareScheduler.`dataTransferTime(java.util.List<File> requiredFiles, Cloudlet cl, int vmId)` |
  - ### Uses of Cloudlet in org.workflowsim.utils

    Methods in org.workflowsim.utils with parameters of type Cloudlet

    | Modifier and Type | Method and Description |
    | --- | --- |
    |  |  |
    | --- | --- |
    | `double` | OverheadParameters.`getClustDelay(Cloudlet cl)` Gets the clustering delay for a particular job based on the depth(level) |
    | `double` | OverheadParameters.`getQueueDelay(Cloudlet cl)` Gets the queue delay for a particular job based on the depth(level) |

- Overview
- Package
- Class
- Use
- Tree
- Deprecated
- Index
- Help

- Prev
- Next

- Frames
- No Frames

- All Classes
